# Effect of integration of supplemental nutrition with public health programmes in pregnancy and early childhood on cardiovascular risk in rural Indian adolescents: long term follow-up of Hyderabad nutrition trial

**DOI:** 10.1136/bmj.a605

**Published:** 2008-07-31

**Authors:** Sanjay Kinra, K V Rameshwar Sarma, Ghafoorunissa, Vishnu Vardhana Rao Mendu, Radhakrishnan Ravikumar, Viswanthan Mohan, Ian B Wilkinson, John R Cockcroft, George Davey Smith, Yoav Ben-Shlomo

**Affiliations:** 1 Non-communicable Disease Epidemiology Unit, London School of Hygiene and Tropical Medicine, London WC1E 7HT; 2 National Institute of Nutrition, Hyderabad 500 007, India; 3 Madras Diabetes Research Foundation, Chennai 600 086, India; 4 Department of Pharmacology, University of Cambridge, Cambridge CB2 2QQ; 5 Welsh Heart Centre, Cardiff CF14 4XN; 6 MRC Centre for Causal Analyses in Translational Epidemiology, Department of Social Medicine, University of Bristol, Bristol BS8 2PR; 7 Department of Social Medicine, University of Bristol, Bristol BS8 2PR

## Abstract

**Objective** To determine whether integration of nutritional supplementation with other public health programmes in early life reduces the risk of cardiovascular disease in undernourished populations.

**Design** Approximately 15 years’ follow-up of participants born within an earlier controlled, community trial of nutritional supplementation integrated with other public health programmes.

**Setting** 29 villages (15 intervention, 14 control) near Hyderabad city, south India.

**Participants** 1165 adolescents aged 13-18 years.

**Intervention** Balanced protein-calorie supplementation (2.51 MJ, 20 g protein) offered daily to pregnant women and preschool children aged under 6 years, coupled with integrated delivery of vertical public health programmes.

**Main outcome measures** Height, adiposity, blood pressures, lipids, insulin resistance (homoeostasis model assessment (HOMA) score), and arterial stiffness (augmentation index).

**Results** The participants from the intervention villages were 14 mm (95% confidence interval 4 to 23; P=0.007) taller than controls but had similar body composition. The participants from the intervention villages had more favourable measures of insulin resistance and arterial stiffness: 20% (3% to 39%; P=0.02) lower HOMA score and 3.3% (1% to 5.7%; P=0.008) lower augmentation index. No strong evidence existed for differences in blood pressures and serum lipids.

**Conclusions** In this undernourished population, integrated delivery of supplemental nutrition with other public health programmes in pregnancy and early childhood was associated with a more favourable profile of cardiovascular disease risk factors in adolescence. This pragmatic study provides the most robust evidence to date on this important hypothesis for which classic trials are unlikely. Improved maternal and child nutrition may have a role in reducing the burden of cardiovascular disease in low income and middle income countries.

## Introduction

Some authors have suggested that the risk of cardiovascular diseases can be “programmed” in early life through the persistence of endocrine, physiological, and metabolic adaptations made in the face of undernutrition.^1^ ^2^ The proposition that inadequate diet in early life may result in heightened sensitivity to lifestyle related risk factors is of some importance to the unfolding cardiovascular disease epidemic in low income and middle income countries, where undernutrition and urbanisation now often coexist.^3^

The evidence in support of this hypothesis is largely circumstantial: animal experiments and observational studies in humans showing associations between anthropometric measures (as proxies for undernutrition) and risk of cardiovascular disease.^1^ ^2^ ^4^ ^5^ Direct evidence of a relation between inadequate diet in early life and later risk of cardiovascular disease is almost non-existent. The importance of balanced protein-calorie reduction has been studied in two natural experiments of starvation and one small randomised controlled trial of nutrition supplementation, but the results have been inconclusive.^6^ ^7^ ^8^ ^9^ We therefore examined the prevalence of risk factors for cardiovascular disease in adolescents born within an earlier community trial of nutritional supplementation offered to pregnant women and young children.^10^

## Methods

Integrated Child Development Services is a national community based programme aimed at improving the health, nutrition, and development of children in India.^11^ ^12^ The centrepiece of this programme is the provision of free food: a cereal based meal prepared variably from locally available ingredients but providing on average about 2.09 MJ and 20-25 g protein to pregnant/lactating women and about 1.25 MJ and 8-10 g protein to children up to 6 years. The supplement has to be collected daily by the woman (or her children) from the Integrated Child Development Services centre (run by a local woman trained for this programme), but they are not obliged to eat it there. To ensure that the impact of food supplementation on the child’s nutritional status is not undermined by ill health, diarrhoea, and frequent infections, the programme is complemented by health, hygiene, and nutrition education for the mothers and delivery of other national programmes (immunisation, anaemia control, and basic health care) from the Integrated Child Development Services centre. These other programmes are available universally (that is, in the control area too), but we anticipate that a common point of delivery increases their uptake in the Integrated Child Development Services programme by making it more convenient.^11^ ^12^ ^13^

### Initial trial design (1987-90)

Using the opportunity afforded by the stepwise expansion of this programme during the 1980s and 1990s, the National Institute of Nutrition in India ran a trial to assess (among other things) the impact of food supplementation in pregnancy on the birth weight of offspring. A cluster of villages with a total population of 30 000 was chosen from each of the two adjacent administrative areas (called “blocks”), one of which already had the Integrated Child Development Services programme in place (intervention arm), whereas the other was awaiting implementation (control arm). As the 100 or more villages in each of the two blocks were spread over an unfeasibly large area, villages were chosen geographically for random selection: contiguous villages falling within a 10 km radius of a prominent central village (deemed to be the approximate centre of the block on visual inspection of the area map) in each block were selected. This process resulted in 15 villages from the intervention arm and 14 villages from the control arm being recruited to the study.

A 12 member team of investigators resided full time in the field for the duration of the study. Following a lead-in period of six months (involving training, pilot data collection, and household enumeration to identify women in the reproductive age group—13-45 years), the trial included all births in the area between 1 January 1987 and 31 December 1990. The “at risk” (of pregnancy) women were monitored monthly to detect missing of menstruation, and those identified as pregnant were followed closely during pregnancy until delivery (with clinical examinations in each of the three trimesters). The field team attempted to visit the home as soon as possible after delivery to collect data on the outcome of the pregnancy and to weigh the newborn within 48 hours. An infant beam balance with a 20 g accuracy (John Chatillon & Sons, NY, USA) was used to measure weight.

The supplement given in this trial was “upma,” a local preparation made from corn-soya blend (120 g) and soybean oil (16 g), providing 2.51 MJ and 20 g protein to the women and half this amount to the children. No other nutrients (such as micronutrients, iodised salt) were added to the supplement. The other universal programmes—immunisation, anaemia control in pregnancy through distribution of iron (60 mg elemental) and folic acid (500 μg) tablets, and the provision of basic health care—existed to a similar extent in both the intervention and control areas, although their uptake may be presumed to have been higher in the intervention area. Crossover of supplements from intervention to control villages was not a problem because of their separation by a large number of villages not involved in the study; in addition, supplements were offered only to the named residents of the village. A preliminary abstract reported the ongoing study,^10^ but full findings were unreported.

### Follow-up survey (2003-5)

We designed this follow-up study to establish the status of women and their offspring who took part in the trial and to clinically examine offspring who were still resident in the area. The parents and children gave written informed consent.

We did two parallel surveys on a village to village basis—the first to identify the study participants and the second to do clinical examinations. The identification survey preceded the clinical survey by three months, and we interchanged the survey area between intervention and control villages every couple of months to even out any variations arising from seasons and experience of the study team. In the identification survey, we first identified women who took part in the baseline trial by using their own and their husband’s name. We interviewed each woman thus identified to collect details on children she had previously borne. All children born in these villages during the initial trial period (1987-90) were potential study participants. However, owing to limited resources and to make maximal use of the baseline data, we invited only those children who could be successfully matched to the previous records to have clinical examinations. We used date of birth and sex for matching, as infants’ names were not recorded in the baseline study (traditionally, naming is delayed and not done at the time of birth in India). Where multiple children of the same woman were eligible, we invited them all and made appropriate adjustments in the analyses.

We used a single clinic site at each village (generally a central point such as the village hall or health centre) to reduce measurement error arising from differences in clinic conditions. We held clinics in the morning, and invited 10-15 children to come fasting each day. We completed a full interviewer administered questionnaire and clinical examination on the children and a brief questionnaire on the mothers (who were asked to accompany the child).

### Measurements

We measured height with a portable stadiometer (Leicester height measure; Chasmors, London). The participant stood erect with his or her head in the Frankfort plane, and a gentle upward pressure was applied under the mastoid. We used a digital weighing machine (HD 305; Tanita, Tokyo, Japan) to measure weight. We measured skinfold thickness at four sites (biceps, triceps, subscapular, and suprailiac) in triplicate with the Holtain calliper (supplied by Chasmors, London). We used a validated oscillometric device (HEM 705; Omron, Matsusaka Co, Japan) to measure blood pressure in the supine position with appropriate cuff sizes; we took two measurements and averaged them for analyses. We measured ambient room temperature with a digital thermometer.

We assessed the augmentation index, a measure of global arterial stiffness, by using an applanation tonometry technique with the Sphygmocor apparatus (Vx system; Atcor (PWV) Medical, Sydney, Australia).^14^ The characteristic pressure waveform produced by blood flow in the arteries changes as the arteries get stiffer with age or under conditions that lead to their premature stiffening (such as atherosclerosis).^15^ Pulse wave analysis can therefore be used to assess the functioning of the vascular tree. Pressure waveforms are obtained non-invasively by applying a pressure sensitive probe over a peripheral artery and transformed into the corresponding central arterial waveform by using a generalised transfer function validated against invasive pressure recordings.^14^ The augmentation index (difference between the first and second peaks of the central arterial waveform, expressed as a percentage of pulse pressure) is negative in healthy young adults but becomes increasingly positive as arteries stiffen.^15^ We measured the augmentation index over the radial artery in the supine position, taking an average of two high quality recordings (quality index >90%).

We classified sexual maturation into four stages (corresponding to Tanner’s early, middle, and late puberty and post-puberty) on the basis of time since the onset of menstruation (girls) and testicular volume (boys).^16^ The boys self assessed testicular volume in private, using Prader’s orchidometer (a chain of 12 wooden testes with volumes ranging from 1 ml to 25 ml). We measured socioeconomic position with the standard of living index, which is a household level, asset based scale devised for use in India.^17^ We classified the participants as having low (0-14), medium (15-24), or high (25-67) socioeconomic position. We assessed urbanisation of the villages by population size (people) and classified them into low (<2000), medium (2000-5000), and high (>5000).

We collected fasting blood samples (at least eight hours) in appropriate vacutainers, transferred them within one to two hours (in icebox at 4-8° C), and processed them within four hours. We did assays for glucose, triglycerides, total cholesterol, and high density lipoprotein cholesterol on the same day with an autoanalyser (ACE Clinical System; Schiapparelli Biosystems, NJ, USA) and the recommended kits (Alfa Wasserman, NJ, USA). We estimated insulin concentrations by radioimmunoassay in batches within four to six weeks.^18^

### Quality control

We produced detailed protocols and used them regularly to standardise the work of the fieldwork team. “Blinding” of the fieldworkers to the group assignment was not an option; however, all measurements except anthropometry were to a large extent automated (for example, biochemical assays, blood pressure, and arterial stiffness), thus reducing the possibility of bias. Only one observer made each measurement (including biochemical assays), to eliminate interobserver bias. We assessed reproducibility of clinic measurements by repeating the measurements on a random subsample (5%) of participants after one to three weeks and found it to be consistently high (intraclass correlation coefficients of >0.98 for anthropometric measures and >0.85 for blood pressures and augmentation index). We put in place internal and external quality control arrangements (with Cardiac Biochemistry laboratory at the All India Institute of Medical Sciences, Delhi, which is part of UKNEQAS system coordinated from Newcastle, UK) for biochemical assays, and split assays were done on a 5% subsample (intraclass correlation coefficients >0.94). We validated testicular self assessment technique against a trained observer in a separate substudy and found it to be highly accurate (mean difference in model ranks (self reported minus directly observed)—0.07 (95% confidence interval −0.11 to 0.25)—with no evidence of systematic bias on a Bland-Altman plot).

### Statistical analyses

We used the log of the sum of four skinfolds to calculate the percentage of body fat^19^ ^20^; we then converted this into fat and fat-free mass by using body weight and expressed them as the corresponding indices after dividing them by the square of height in metres.^21^ We assessed central adiposity by the ratio of central (subscapular plus suprailiac) to peripheral (biceps plus triceps) skinfolds. We estimated low density lipoprotein cholesterol from triglycerides, total cholesterol, and high density lipoprotein cholesterol by using the Friedewald-Fredrickson equation,^22^ and we calculated insulin resistance by homoeostasis model assessment (HOMA), excluding those with fasting glucose ≥7 mmol/l.^23^

We applied suitable transformations to outcome variables that deviated markedly from a normal distribution (triglycerides, insulin, and HOMA scores). We used linear regression models to investigate association of supplemental nutrition with cardiovascular disease risk factors. Analysis was on an intention to treat basis, using area of birth as proxy (irrespective of whether the participant took the supplement or not). We fitted four predefined models to adjust incrementally for the main domains of potential confounding or intermediary variables: model 1 (physiological variables—age, sex, pubertal stage); model 2 (current socioeconomic status—household standard of living index, village population); model 3 (stature—height); and model 4 (body composition—fat mass index, fat-free mass index, and central-peripheral skinfold ratio). We additionally adjusted blood pressure for ambient room temperature and augmentation index for heart rate, as these factors can artefactually affect their values.^24^ ^25^ We assessed association of supplemental nutrition with sexual maturation (by ordinal and binary logistic regression), as improved nutrition may influence risk of cardiovascular disease through earlier onset of puberty.^26^

Data could be clustered at the level of the village and the household (in case of multiple children). To take account of village level clustering, we used robust standard errors in all the models, with village as the level of cluster. This technique uses the cluster level residuals to derive the standard errors, so that the resulting standard errors are larger and valid in the presence of clustering but the parameter estimate remains unchanged. This technique was inappropriate for household level clustering, as very few households had multiple children taking part in the study. We therefore examined the impact of household level clustering on the results by excluding the second of the two children from the same household (no household contained more than two children). We similarly estimated the impact of a few migrant children (who usually lived outside the area but happened to be at home during the study) on the results by excluding these children from the final models. We examined interaction between the intervention and the sex of the participant, as evidence exists of preferential feeding of male children in this setting,^27^ as well as possible sex differential effects of early undernutrition on risk of cardiovascular disease.^28^ To retain maximum study power, we examined only one interaction. We used Stata version 9 for statistical analyses.

Sample size calculations done before the start of the study suggested that the anticipated sample (estimated as 1268 overall) was going to be adequate to detect important differences in most outcomes (for example, at 80% power, 5% significance level, and 0.01 intraclass correlation coefficient for village level clustering, the mean detectable differences were 1.8-2.9 mm Hg for systolic blood pressure, 1.5% for augmentation index, and 1.7-3.2 mU/l for insulin).

## Results

Of the 4338 pregnancies recorded in the trial, birth weights (recorded within 48 hours) were available for 2964 (68%) children. The mean birth weight of children born in the intervention area (2655 (SD 424) g) was higher than that of controls (2594 (SD 430) g); the mean difference was 61 g (95% confidence interval 18 to 104; P=0.007). Adjustment for sex of the child made no difference to the results; however, we deemed data on gestational age to be of insufficient quality (strong digit preference) to be included in the analyses. Personal information (necessary to trace families) was available for 2756 women, of which 1963 (71%) could be contacted successfully (figure[Fig fig1]). At the time of follow-up, the contacted women had delivered 8246 children, of which 2601 were eligible for follow-up (born between 1987 and 1990 and still alive in 2003). From these eligible children, we invited only those with existing information in the trial dataset (n=1492; 57%) to have a clinical examination. A total of 1165 children participated in the clinics: 654 (82%) in the intervention area and 511 (74%) in the control area, representing 45% of all eligible births from the area at the time. Children who took part in the clinics were slightly older and more likely to be males and students than those who were eligible but did not participate (table 1).

**Figure fig1:**
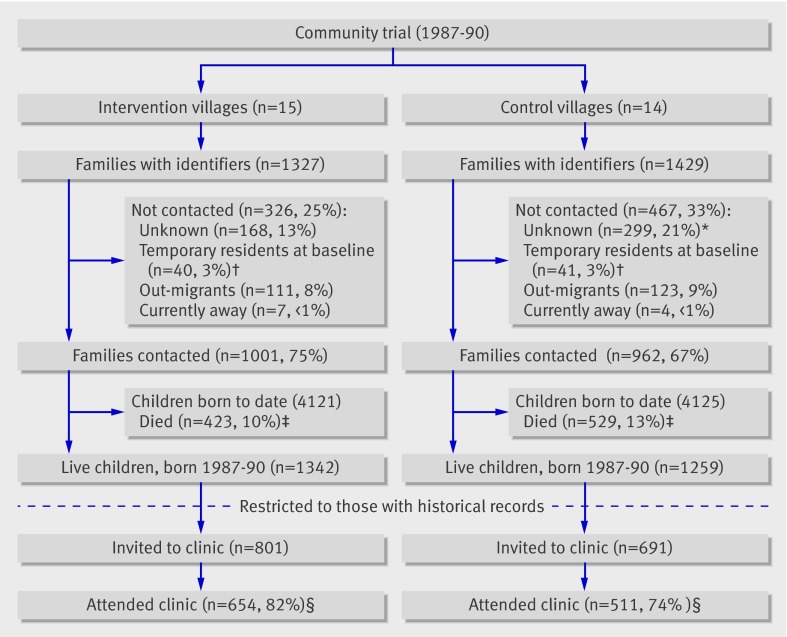
Flow chart of participant recruitment at follow-up in Hyderabad nutrition trial. *Higher figure in control area probably reflects greater influx of temporary migrant workers during harvest season. †Married daughters of villagers who were temporarily visiting their parents’ home for childbirth at time of baseline study. ‡Data are for all children (rather than study cohort), as date of birth of dead children was not collected (deemed insensitive). §These represent 49% (intervention) and 41% (control) of all eligible births

**Table 1 tbl1:** Characteristics of children who attended and those who did not attend clinical examination at follow-up in Hyderabad nutrition trial. Values are numbers (percentages) unless stated otherwise

Characteristic	Intervention area (n=1342)				Control area (n=1259)		
	Clinic attenders (n=654)	Clinic non-attenders (n=688)	P value*		Clinic attenders (n=511)	Clinic non-attenders (n=748)	P value*
Mean (SD) age (years)	15.5 (0.9)	15.1 (1.2)	<0.001		15.7 (0.9)	15.3 (1.2)	<0.001
Male	349 (53)	338 (49)	0.12		279 (55)	355 (47)	0.013
Occupation:	(n=653)	(n=686)	0.001			(n=745)	0.012
Full time student	529 (81)	496 (72)			372 (73)	507 (68)	
Full time employment	84 (13)	126 (18)			96 (19)	194 (26)	
Others (neither, both)	40 (6)	64 (9)			43 (8)	44 (6)	

*Based on unpaired *t *tests or χ^2^ tests for heterogeneity with appropriate degrees of freedom.

P values for interaction tests between intervention area and clinic attendance: age (P=0.4), male sex (P=0.5), and occupation (P=0.07).

Three girls who reported being pregnant were excluded from the analyses. Of the remaining 1162 children, we excluded those with missing data on key confounders and, for biochemical parameters only, those who had fasted for less than eight hours. Finally, complete data were available for 1120 (96%) children for at least one outcome, with slightly fewer for blood pressure (1118; 96%), lipid profile (1050; 90%), glucose-insulin (1008; 87%), and arterial stiffness (862; 74%). The lower number with glucose-insulin data was due to a failure to obtain insulin assay reagent in time for the last batch of assays (n=48), and that for arterial stiffness data was due to an inability to complete all assessments during the course of a clinic day, as most villages lacked the continuous electricity supply needed to run the apparatus. Some, but not all, of the participants returned subsequently to have their assessments completed.

Table 2 shows the distributions of key exposures, which were largely similar across the two areas. The distribution of the standard of living index was consistent with the peri-urban situation of these villages. Only two children reported smoking tobacco or consuming alcohol, and all except two were breast fed, so we did not consider these variables in further analyses. Table 3 shows the data for the main outcome measures by the area of intervention. The distributions of triglycerides, insulin concentration, and HOMA score were positively skewed but log-normal. We therefore present geometric means and logged regression coefficients for these. Body composition data confirm the short, lean habitus of this population. The mean values for indices of insulin resistance (insulin and HOMA score) and, to a smaller extent, arterial stiffness (although data are limited) were higher than those generally reported from high income countries, suggesting a pro-atherogenic profile in this undernourished population.^29^ ^30^ The average number of participants from each village was 40 (range 2-122). Village level clustering of outcome measures was less than 0.1, except for fasting blood glucose (0.14). All results are based on models with robust standard errors, with village as the level of cluster.

**Table 2 tbl2:** Distribution of key exposures in participants who completed clinic questionnaires at follow-up in Hyderabad nutrition trial. Values are numbers (percentages) unless stated otherwise

Characteristic	Intervention area (n=633)	Control area (n=498)	P value*
Mean (SD) age (years)	15.8 (0.9)	15.9 (0.9)	0.02
Male	333 (53)	274 (55)	0.4
Pubertal stage†:	(n=628)	(n=496)	0.4
Early puberty	88 (14)	83 (17)	
Middle puberty	209 (33)	156 (31)	
Late puberty	184 (29)	132 (27)	
Post-puberty	147 (23)	125 (25)	
Standard of living index:	(n=630)		0.2
High (25-67)	228 (36)	158 (32)	
Medium (15-24)	291 (46)	239 (48)	
Low (0-14)	111 (18)	101 (20)	
Own occupation‡:		(n=497)	0.002
Student/vocational training	534 (84)	391 (79)	
Employed	66 (10)	53 (11)	
Unemployed/housework	33 (5)	53 (11)	
Literate mother§	58 (9)	57 (11)	0.2
Lifestyle:		(n=497)	
Consumed tobacco (ever)	0	2 (0)	0.2
Consumed alcohol (ever)	0	2 (0)	0.2

*P values are based on unpaired *t *tests or χ^2^ tests for heterogeneity with appropriate degrees of freedom.

†Pubertal stage for boys: intervention area—early 27%, middle 56%, late 18%, post-puberty 0%; control area—early 30%, middle 50%, late 19%, post-puberty 0%. Pubertal stage for girls: intervention area—early 0%, middle 8%, late 42%, post-puberty 50%; control area—early 0%, middle 9%, late 35%, post-puberty 56%.

‡Vocational training, n=15; unemployed, n=36.

§Literate/primary school, n=45; middle school, n=43; high school, n=16; intermediate level, n=9; graduate, n=2.

**Table 3 tbl3:** Distribution of outcome variables by area of intervention at follow-up in Hyderabad nutrition trial

	No	Mean (SD)		Mean difference* (95% CI) (control minus intervention)
		Intervention	Control	
**Body size and composition**				
Height (mm)	1120	1559 (83)	1549 (82)	−10.0 (−18.7 to −1.4); P=0.024
Body mass index (kg/m^2^)	1120	17.1 (2.0)	17.3 (2.4)	0.27 (−0.08 to 0.61); P=0.1
Fat mass index (kg/m^2^)	1120	2.6 (1.3)	2.6 (1.5)	−0.02 (−0.23 to 0.19); P=0.8
Fat-free mass index (kg/m^2^)	1120	14.5 (1.4)	14.8 (1.6)	0.29 (0.01 to 0.57); P=0.043
Central-peripheral skinfold ratio	1120	1.48 (0.25)	1.47 (0.25)	−0.01 (−0.06 to 0.05); P=0.8
**Cardiovascular physiology**				
Systolic blood pressure (mm Hg)	1118	108.7 (10.3)	109.6 (10.0)	0.83 (−1.44 to 3.11); P=0.5
Diastolic blood pressure (mm Hg)	1118	62.5 (6.5)	62.2 (6.5)	−0.23 (−1.70 to 1.25); P=0.8
Augmentation index (%)	862	2.5 (11.4)	5.6 (9.1)	3.16 (0.8 to 5.51); P=0.011
**Lipid profile**				
Total cholesterol (mmol/l)	1050	3.45 (0.69)	3.45 (0.67)	−0.00 (−0.12 to 0.12); P=1.0
LDL cholesterol (mmol/l)	1050	2.05 (0.60)	2.04 (0.59)	−0.02 (−0.13 to 0.09); P=0.8
HDL cholesterol (mmol/l)	1050	0.99 (0.23)	1.00 (0.22)	0.01 (−0.05 to 0.06); P=0.7
Triglycerides† (mmol/l)	1050	0.82 (0.39 to 1.72)	0.83 (0.40 to 1.74)	0.02 (−0.04 to 0.07); P=0.6
**Glucose homeostasis**				
Glucose (mmol/l)	1008	4.68 (0.58)	4.72 (0.74)	0.03 (−0.23 to 0.29); P=0.8
Insulin† (mU/l)	1008	15.36 (5.07 to 46.52)	18.45 (6.37 to 53.41)	0.18 (0.03 to 0.33); P=0.02
HOMA score†	1003	3.16 (1.00 to 10.00)	3.79 (1.22 to 11.73)	0.18 (0.04 to 0.32); P=0.015

HDL=high density lipoprotein; HOMA=homoeostasis modal assessment; LDL=low density lipoprotein.

*Based on linear regression models with robust standard errors.

†Values for intervention and control areas are geometric means and 95% reference ranges; mean differences are on log scale.

Children in the intervention and control arms were not different in their sexual maturation (data not shown). Table 4 shows the results of multivariably adjusted associations between supplemental nutrition and risk factors for cardiovascular disease. Children in the intervention area were on average 14 (95% confidence interval 4 to 23) mm taller than the control children but similar in body composition. Systolic blood pressure (but not diastolic blood pressure) was slightly higher in the control area, although the difference did not reach conventional levels of statistical significance. The augmentation index showed an association with supplemental nutrition, which was unlikely to be due to chance and was hardly altered by multivariable adjustments; higher values (stiffer arteries) were seen in the control area. Lipids and glucose were not associated with supplemental nutrition. However, children in the control arm had higher insulin concentration and HOMA score, robust to adjustments for height and body composition (identical results for insulin and HOMA due to high correlation). The regression coefficients shown in table 4 for insulin and HOMA score are differences between the means on the log scale; the HOMA score value for model 2 equates to 20% (95% confidence interval 3% to 39%) higher values in the control children (on the original scale). We found little evidence to support a sex differential effect of nutritional supplementation (data not shown). Limiting the analyses to children with information on augmentation index or HOMA score did not change the results for other outcomes materially.

**Table 4 tbl4:** Multivariable association* between supplemental nutrition and cardiovascular disease risk factors at follow-up in Hyderabad nutrition trial

Measure	β coefficient (95% CI); P value			
	Model 1†	Model 2‡	Model 3§	Model 4¶
Height (mm)	−14.1 (−23.3 to −4.9); 0.004	−13.6 (−23.1 to −4.1); 0.007	NA	NA
Fat mass index (kg/m^2^)	0.01 (−0.15 to 0.16); 0.9	0.04 (−0.10 to 0.18); 0.5	0.06 (−0.07 to 0.19); 0.4	NA
Fat-free mass index (kg/m^2^)	0.23 (0.03 to 0.43); 0.029	0.04 (−0.10 to 0.18); 0.5	0.28 (0.11 to 0.46); 0.003	NA
Central-peripheral skinfold ratio	−0.02 (−0.06 to 0.03); 0.5	−0.02 (−0.08 to 0.03); 0.4	−0.01 (−0.07 to 0.05); 0.7	NA
Systolic blood pressure (mm Hg)	0.86 (−0.72 to 2.45); 0.2	0.59 (−1.11 to 2.29); 0.4	1.10 (−0.68 to 2.87); 0.2	0.64 (−0.99 to 2.27); 0.4
Diastolic blood pressure (mm Hg)	0.21 (−0.81 to 1.23); 0.7	0.08 (−0.90 to 1.07); 0.8	0.21 (−0.77 to 1.19); 0.6	0.11 (−0.85 to 1.07); 0.8
Augmentation index (%)	3.29 (0.74 to 5.84); 0.013	3.30 (0.96 to 5.65); 0.008	2.84 (0.39 to 5.30); 0.025	2.96 (0.55 to 5.38); 0.018
Total cholesterol (mmol/l)	0.01 (−0.11 to 0.12); 0.9	−0.02 (−0.11 to 0.08); 0.7	−0.02 (−0.12 to 0.07); 0.6	−0.03 (−0.13 to 0.06); 0.5
LDL cholesterol (mmol/l)	−0.01 (−0.12 to 0.09); 0.8	−0.03 (−0.13 to 0.07); 0.6	−0.03 (−0.13 to 0.07); 0.5	−0.04 (−0.14 to 0.06); 0.4
HDL cholesterol (mmol/l)	0.01 (−0.05 to 0.06); 0.7	−0.00 (−0.06 to 0.05); 1.0	−0.00 (−0.06 to 0.05); 0.9	−0.00 (−0.06 to 0.05); 0.9
Triglycerides (mmol/l)**	0.02 (−0.03 to 0.07); 0.4	0.03 (−0.03 to 0.08); 0.3	0.02 (−0.03 to 0.08); 0.4	0.02 (−0.03 to 0.07); 0.4
Glucose (mmol/l)	0.04 (−0.22 to 0.29); 0.8	0.03 (−0.21 to 0.26); 0.8	0.03 (−0.21 to 0.26); 0.8	0.04 (−0.20 to 0.28); 0.8
Insulin (mU/l)**	0.18 (0.04 to 0.32); 0.016	0.18 (0.03 to 0.34); 0.02	0.19 (0.04 to 0.35); 0.016	0.19 (0.03 to 0.35); 0.016
HOMA score**	0.18 (0.04 to 0.32); 0.014	0.18 (0.03 to 0.33); 0.021	0.19 (0.04 to 0.35); 0.016	0.19 (0.03 to 0.34); 0.02

HDL=high density lipoprotein; HOMA=homoeostasis model assessment; LDL=low density lipoprotein; NA=not applicable.

Sample size: n=1120 for height, fat mass index, fat-free mass index, and central-peripheral skinfold ratio; n=1118 for systolic and diastolic blood pressure; n=862 for augmentation index; n=1050 for total cholesterol, HDL cholesterol, LDL cholesterol, and triglycerides; n=1008 for glucose and insulin; n=1003 for HOMA score.

*Linear regression models with robust standard errors.

†Adjusted for age, sex, pubertal stage (early puberty, middle puberty, late puberty, and post-puberty), room temperature (blood pressure only), and heart rate (augmentation index only).

‡Adjusted for variables in model 1 plus current socioeconomic circumstances (household’s standard of living index (high, medium, low) and village urbanisation (village population <2000, 2000-5000, >5000)).

§Adjusted for variables in model 2 plus height (mm).

¶Adjusted for variables in model 3 plus body composition (fat mass index, fat free mass index, and central-peripheral skinfold ratio).

**Differences (between means) are on log scale.

The study included 36 sibling pairs, of which five pairs were twins; all other households contributed only one child to the study. Thirty two participants had migrated away from their place of birth, of which nine had migrated within the study area (all except one had migrated within their own category of area—that is, intervention or control). Exclusion of the migrant children or the second child (in the case of sibling pairs) made no material difference to the results.

## Discussion

This is the first intervention study to show that modest improvements in the protein-calorie intake of pregnant women and young children may result in a more favourable cardiovascular disease risk factor profile among populations with prevalent undernutrition. These findings may indicate potential life course pathways underlying the causes of cardiovascular diseases in general.^31^ These observations, if replicated, have important implications for the primary prevention of cardiovascular diseases in low income and middle income countries, as the intervention tested was pragmatic, cheap, and relatively easy to implement.

### Comparison with previous research

The importance of balanced protein-calorie malnutrition in early life has been studied in two natural experiments based on the starvation experience of populations under siege in the second world war (Dutch and Leningrad studies) and one small randomised controlled trial of supplemental nutrition from Guatemala.^6^ ^7^ ^8^ ^9^ Whereas the Dutch study reported associations with adiposity and dyslipidaemia (early gestation starvation) and with abnormal glucose homoeostasis (late gestation starvation), these findings were not replicated in the Leningrad study.^7^ ^8^ The Guatemala trial randomised four villages within pairs to offer either low energy (1.38 MJ) or high energy (3.76 MJ, proteins, and micronutrients) supplements to pregnant women and children until the age of 7 years.^6^ ^9^ Follow-up at age 24 years suggested no association of supplement type with adiposity or blood pressure but an association of higher energy supplement with lower fasting glucose in women only. One potential reason for the negative findings could be that these studies were underpowered, with sample sizes of 741 (68 in early gestation, 120 in late gestation starvation categories) in the Dutch study, 549 (169 with intrauterine starvation) in the Leningrad study, and 450 (167 women) in the Guatemala study. Alternatively, the exposure may have been inadequate for programming of long term physiological changes, in terms of either strength (mean birth weights in all three studies were greater than 3.0 kg) or duration (the two natural experiments).

### Strengths and limitations

The main strength of this study is its setting: a nutritional intervention in a population with high background levels of chronic undernutrition, so we could realistically expect to find programming effects of inadequate diet, if they exist. The controlled design of the original evaluation would have reduced the chances of confounding; furthermore, the crucial confounders were either completely absent (maternal smoking) or severely restricted (socioeconomic heterogeneity and urbanisation, with the consequent heterogeneity in diet and physical activity behaviour).^32^ The age group (adolescence) was also ideal for studying exposures in early life, as the participants were old enough to show variations in risk factors but young enough that any associations seen were not distorted by any differences in adult lifestyle in the intervening years.

The study also has some important limitations that need to be acknowledged, chief among them the potential for bias owing to non-randomisation of villages in the baseline study, losses to follow-up, and lack of data on current diet and patterns of physical activity. In the baseline study, all villages within a radius of the central village were selected in both intervention and control areas, making selection bias unlikely. Bias could arise, however, if the intervention area urbanised at a different rate from the control area, thus influencing the prevalence of risk factors for cardiovascular disease. Data on urbanisation (such as population size and density; proportion of households with television; and availability of facilities such as electricity, bank, and health centre) collected from the village heads showed little variation between the villages (data not shown), and adjustment for urbanisation (population size) did not materially alter any of the results. Areas considered deprived are prioritised for the introduction of the Integrated Child Development Services programme in phases; however, the staggered introduction of the programme in similarly deprived and adjacent areas (such as those in the study) is mainly due to financial and operational constraints associated with the introduction of a public programme in a large country (about 75% of the villages have been covered in the past 30 years or so, and the programme is still being rolled out).^11^ ^12^ The introduction of the Integrated Child Development Services programme in control areas started in 1992-3, as a result of which some children could have received the supplement when they were 3-6 years old, potentially diluting the effect estimate. However, such programmes take time to get established, with substantial uptakes only after several years, rendering this bias unimportant. The programme has continued since its introduction in both the intervention and control areas, but children over the age of 6 cannot use its services as they are not beneficiaries in the programme.^11^ ^12^

The response rate among children invited to the clinic was high (78%), although it was somewhat lower in the control areas, which in all probability arose from lack of interest in participation in the study shown by heads of some of the control villages (as local elections were taking place at the time). Loss to follow-up resulting from this is likely to be non-systematic. The participating children represented 45% of all eligible births in the area from that time, which compares favourably with the overall follow-up rates in other relevant studies (all below 35%).^6^ ^7^ ^8^ ^9^ Comparisons of participating children with the remaining children who were eligible but did not participate (using limited available information) showed them to be little different. The most important reason for failing to trace all eligible participants, we believe, was the inclusion of temporary migrant workers in the initial study, as such participants would not have remained in the village at the time of the follow-up study. Unfortunately, this information was not recorded in the initial trial. Lack of this and other baseline data as a result of limited matching precluded any meaningful use of sophisticated techniques for handling missing data (such as imputation). Bias in the results due to incomplete follow-up cannot be ruled out, so the results should be interpreted with some caution.

Differences in current diet and patterns of physical activity could account for the differences seen in risk of cardiovascular disease. Although it is theoretically possible, we see no obvious reason to believe that children from these neighbouring villages had marked differences in lifestyle. The villages are still fairly homogeneous in their diet (limited range of foods eaten mostly at home) and activity patterns (generally active). Furthermore, no differences in body composition existed; as adiposity reflects the net energy balance between intake (diet) and expenditure (physical activity), the low fat mass in this population and its narrow distribution range, argues against any important lifestyle differences between the two areas. Finally, despite the relative automation of most of the outcome measures used in this study (including insulin resistance and arterial stiffness), the possibility of bias arising from the lack of blinding of the fieldworkers cannot be ruled out completely.

### Potential mechanisms

Insulin resistance is believed to be central to many of the changes attributed to the thrifty phenotype.^4^ ^33^ If the fetus develops insulin resistance in times of undernutrition, this may improve the short term chances of survival but predispose to cardiovascular disease on persistence into adult life.^34^ ^35^ ^36^ ^37^ Arterial stiffness is a relatively novel risk factor for atherosclerosis.^38^ Whether it is a risk marker for atherosclerosis or an independent risk factor for cardiovascular disease is unclear.^38^ ^39^ ^40^ However, various other programming pathways are believed to culminate in a self perpetuating cycle of increasing blood pressure and arterial stiffness, which promotes atherosclerosis.^38^ ^39^ Insulin is a risk factor for arterial stiffness and may be the link between early undernutrition, arterial stiffness, and hypertension.^41^ ^42^

The relative importance of nutrition in various stages (pregnancy, infancy, early childhood), sources of nutrition, or indeed the rate of growth in early versus late postnatal life cannot be delineated from this study, as the intervention was given throughout.^43^ The other public health programmes (such as health education and immunisation) were available in both intervention and control areas, but they may have had greater uptake in the intervention area owing to their integration with the nutritional component. Data on the uptake of these components were not available, so their independent effects on the results cannot be ruled out.^44^ However, evidence suggests that their contribution to cardiovascular disease risk, if any, is more likely to have been mediated through improvements in the nutritional status, by helping to break the vicious cycle of infections, diarrhoea, and malnutrition found in these settings.^45^

### Public health implications

We need to contextualise our observations and consider what public health benefits may accrue if this intervention was translated into primary prevention of disease burden. The Anglo-Cardiff collaborative study suggested that a 3% lower augmentation index equates to a three year less aged vascular phenotype.^29^ Given the age of the study population, this would be a substantial gain; however, extreme caution in interpretation is warranted because of differences in study setting and design. Similarly, extrapolating the data from a meta-analysis of studies on insulin concentrations and risk of cardiovascular disease also suggests a potential 8% reduction in cardiovascular disease risk of the supplemented children.^36^ Although these improvements are substantial in themselves, even more important is the potential for these risk factors to interact with the behavioural risk factors in adulthood, resulting in a major amplification in the risk difference.^1^ ^34^

The intervention in this study was given within the framework of a public welfare programme.^11^ ^12^ In such programmes, the supplement is collected by only about half of the eligible women and often shared among other family members; the study therefore probably underestimates the potential benefit. On the other hand, the effects of dietary supplements on the nutritional status of the child were probably augmented by other components of the programme, such as health education and immunisation. This study therefore provides realistic estimates of the benefit that can be expected from supplemental nutrition given in the context of “holistic” programmes to tackle undernutrition in children. Such programmes already exist around much of the developing world,^46^ but their advisability is being questioned in view of the epidemics of obesity and cardiovascular disease.^47^ For example, a previous cohort study from India found that risk of diabetes and impaired glucose tolerance was associated with thinness at age 2 years and increased adiposity at 12 years.^48^ Our results provide some reassurance about the longer term benefits of improving maternal and childhood nutrition in undernourished populations. At this stage in the participants’ life course, this intervention does not seem to increase the risk of obesity and may even decrease (not increase) the future risk of cardiovascular disease.

**What is already known on this topic**

Observational studies suggest that early undernutrition predisposes to cardiovascular disease in later life, but robust evidence from intervention trials is lacking

**What this study adds**

Integration of supplemental nutrition with public health programmes in pregnancy and early childhood was associated with a reduction in cardiovascular risk
Improved maternal and child nutrition may have a role in reducing the burden of cardiovascular disease in low income and middle income countries

## Notes

We acknowledge the institutional support provided by the National Institution of Nutrition, Hyderabad (B Sivakumar, Director). We thank Pete Shiarly for support in data management, Roger Harbord for statistical advice, Shah Ebrahim for comments on an earlier draft of this paper, and Umesh Kapil for advice on the functioning of the Integrated Child Development Services programme. Finally, we are extremely grateful to our committed and diligent fieldwork team (Mrs Jaya Sudha, Mr Krishna Kanth, Mrs Radha, Mr Srinivas, Mrs Jalajja, and Mr Rama Rao) and the study participants without whom this study would not have been possible.

Contributors: SK and KVRS conceived the study and designed it together with YB-S, GDS, and Gh. SK and KVRS implemented the fieldwork and trained and supervised the field teams. RR, VM, IBW, and JRC contributed to the design and training related to arterial stiffness measurements; Gh trained the biochemist and supervised the laboratory assays; and MVVR contributed to data management. SK did the statistical analyses and wrote the first draft of the paper. All authors contributed to writing the manuscript and saw and approved the final version. SK is the guarantor.

Ethics approval: Ethics committee of the National Institute of Nutrition of India, Hyderabad. Approval was also sought from the village heads and their committees in each of the 29 villages.

Provenance and peer review: Not commissioned; externally peer reviewed.
